# Direct synthesis of hydrogen fluoride-free multilayered Ti_3_C_2_/TiO_2_ composite and its applications in photocatalysis

**DOI:** 10.1016/j.heliyon.2023.e18718

**Published:** 2023-07-27

**Authors:** Tao Wang, Li Zhu, Wanying Zhu, Hideki Kanda

**Affiliations:** Department of Materials Process Engineering, Nagoya University, Nagoya, 464-8603, Japan

**Keywords:** Ti3C2 MXene, (NH4)2TiF6, TiO2, Hybrids, Etching, Photocatalytic degradation

## Abstract

Ti_3_C_2_/TiO_2_ hybrids are environment-friendly and exhibit excellent photocatalytic and hydrogen-generating power characteristics. Herein, a novel single-step method is proposed for fabricating multilayer structures in which TiO_2_, generated from (NH_4_)_2_TiF_6_, wraps the Ti_3_C_2_ MXene by etching Ti_3_AlC_2_ with (NH_4_)_2_TiF_6_. The optimal reaction conditions for the etching of Ti_3_AlC_2_ with (NH_4_)_2_TiF_6_ were systematically studied. The phase composition, morphology, and photophysical properties of the Ti_3_C_2_/TiO_2_ hybrids were investigated using X-ray diffraction, field-emission scanning electron microscopy, energy-dispersive X-ray spectroscopy, transmission electron microscopy, X-ray photoelectron spectroscopy, Raman spectroscopy, and UV–vis spectrophotometry. The thermal stability of the hybrids was investigated using thermogravimetric and differential thermal analyses. Along with the formation of Ti_3_C_2_ MXene, Ti_3_AlC_2_ reacted with (NH_4_)_2_TiF_6_ at 60 °C for 24 h to form hybrids surrounded by NH_4_TiOF_3_ crystals. Subsequent reactions of these hybrids with H_3_BO_3_ resulted in the conversion of NH_4_TiOF_3_ crystals into TiO_2_ and eventually into Ti_3_C_2_/TiO_2_ hybrids. Furthermore, the photocatalytic activity of the Ti_3_C_2_/TiO_2_ hybrids was measured by monitoring the photodegradation of methylene blue under ultraviolet light, which showed that the photocatalytic activity of the Ti_3_C_2_/TiO_2_ hybrids was higher than that of the commercial anatase TiO_2_ nanoparticles.

## Highlights

• A novel synthesis of Ti_3_C_2_ MXene using (NH_4_)_2_TiF_6_ as an etching agent.
• TiO_2_ formed from (NH_4_)_2_TiF_6_ can be hybridized with Ti_3_C_2_ MXene.
• This method is hydrofluoric acid-free, and the composite has a fine structure.
• The composite exhibited good photocatalytic performance.

## Graphical abstract

**Figure:**
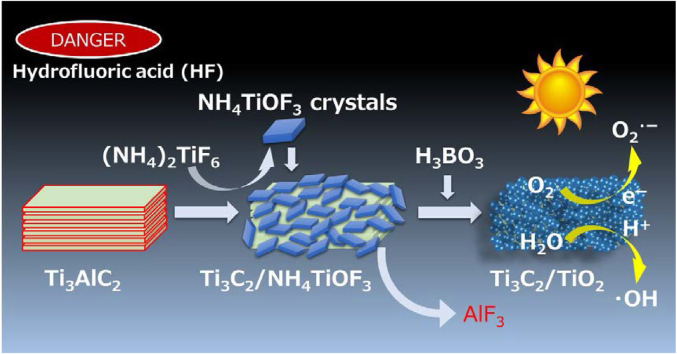

## 1 Introduction

Since the discovery of graphene in 2004, research on two-dimensional (2D) solids has rapidly expanded worldwide owing to the unique properties of graphene offered by its low dimensionality [1,2]. Simultaneously, several scientists have attempted to synthesize 2D materials such as MXene, which is composed of C and transition metals such as Ti and V, as materials beyond graphene for applications in electronic, photonic, and energy storage devices [3].

The catalytic use of light energy has been extensively explored as a potential solution for solving the energy, resource, and environmental problems facing the chemical industry worldwide and has gained significance due to the worsening of pollution [4]. Photocatalytic oxidation is an environmentally friendly technology that is expected to have a wide range of applications in the field of environmental protection. It can be used to decompose organic pollutants present in wastewater and the atmosphere under normal temperature and pressure conditions owing to its ability to utilize direct sunlight [5]. Semiconductors and metal complexes are used as photocatalysts, among which TiO_2_ is the most popular owing to its lower toxicity, higher activity, and better chemical stability than other metal-oxide photocatalysts [6]. Elemental Ti, which makes up TiO_2_, is the ninth most abundant element in the Earth's crust and is found in ilmenite, rutile, and anatase ores. TiO_2_ is a harmless and chemically stable white-colored inorganic compound that is characterized by its significantly high refractive index, dielectric constant, and insulation resistance. The refractive indices of TiO_2_ are 2.72 and 2.52 for the rutile and anatase ores, respectively; thus, TiO_2_ exhibits the largest refractive index among minerals that transmit visible light [7]. Since the Honda–Fujishima effect of water photolysis using TiO_2_ electrodes was first reported in Nature in 1972 [8], several studies have been conducted focusing on the oxidizing power of TiO_2_ as a photocatalyst [9]. The core of photocatalytic oxidation technology lies in the development of highly efficient photocatalysts.

The photoactivity of TiO_2_ allows for controlling its morphology via the synthesis of hybrids and improving and fine-tuning key properties, such as crystalline phase, surface area, exposed crystalline surfaces, surface non-coordination sites, lattice defects, and crystallinity [10]. In addition, the formation of heterojunctions between TiO_2_ and other materials allows TiO_2_ to promote charge separation and visible light absorption by the added materials [11]. TiO_2_ applications require co-catalysts that play a role in stabilizing the photoexcited electrons and holes; these co-catalysts are also important for charge storage [12]. Carbon is considered a promising adsorbent that is capable of concentrating organic substances decomposed by hydroxyl radicals (OH). These radicals are photogenerated by TiO_2_ on the surface of the complex [13]. However, the development and performance evaluation of carbon/TiO_2_ hybrids have been primarily focused on the hydrothermal growth or solvothermal synthesis of TiO_2_ nanostructures [14], graphene oxide [15], and sol-gel techniques [16]. Nanohybrids of Ti_3_C_2_ atom-layered compounds and TiO_2_ have been receiving significant attention in the last few years. A good electron mobility of Ti_3_C_2_ and synergistic effect of TiO_2_ photocatalysis are expected [17]. However, TiO_2_ is obtained as a result of the thermal sintering reaction of Ti_3_C_2_; therefore, it may promote the recombination of electrons and holes in TiO_2_ because of oxygen scarcity, which may degrade its photocatalytic function [18].

MXene is a novel family of 2D transition metal carbides, carbonitrides, and nitrides that was discovered and developed by a research team at Drexel University in 2011 [19,20]. Among the MXene family, Ti_3_C_2_ MXene was the first and most studied MXene. The Ti_3_C_2_ MXene has been studied for its applicability in various fields, such as conductive materials [21], hydrogen production [22], CO_2_ reduction [23], supercapacitors [24], solar cells [25], and lithium-ion batteries [26], owing to its excellent conductivity, thermal conductivity, toughness, chemical resistance, and heat resistance. Furthermore, MXene exhibits significantly better light-to-heat conversion efficiency [27], electromagnetic, and shielding than conventional photothermal materials such as graphene, owing to its large surface area [28]. In some cases, MXene-rGO hybrids perform better than graphene and MXene standalone materials [28,29]. Typically, MXene is obtained as a multilayered powder or as detached single flakes. During the synthesis of MXene powder, a fluoride-containing etchant (HF 50 wt%) is generally used to selectively remove the Al layer [19]. The Ti–Al bond is more fragile than the Ti–C bond. The –F and –OH functional groups are formed on the surface of MXene, resulting in a delaminated layered product. However, the use of HF solution (50 wt%) in the preparation of MXene is limited by its high toxicity and generation of putrefactive effluents. Therefore, it is particularly important to find alternative, non-hazardous synthetic methods for the stable synthesis of MXenes. Various MXene etching methods have been reported, including NaBF_4_–HCl [30], LiF–HCl [31], NH_4_HF_2_–polar organic solvents [32], NH_4_F–HCl [33], Lewis acidic molten salts, such as ZnCl [34] and CuCl_2_ [35], ultrasound-assisted etching [36], and hydrothermal methods [37]. Halim reported that Ti_3_C_2_Tx thin films can be easily produced via room-temperature etching of epitaxial Ti_3_AlC_2_ thin films in HF or NH_4_HF_2_ solutions [38]. Following this pioneering study, Feng et al. reported the synthesis of 2D MXene Ti_3_C_2_ using NH_4_HF_2_ instead of HF and etched the Ti_3_AlC_2_ powder synthesized by non-pressure sintering [39]. The effects of time, temperature, and diameter of Ti_3_AlC_2_ particles on the etching process were determined. 2D MXene Ti_3_C_2_ with a large interplanar spacing was successfully synthesized by etching Ti_3_AlC_2_ with bifluorides (NaHF_2_, KHF_2_, and NH_4_HF_2_) in a single-stage process [40].

The liquid-phase deposition (LPD) method yields stable fluoride complexes in the hydrolytic equilibrium reaction of metal fluoride complex ions (e.g., TiF_6_^2−^ and SiF_6_^2−^). This is a film-forming method wherein the oxides or oxyhydroxides are uniformly deposited and grown on an aqueous solution by adding a fluoride ion scavenger, such as metallic Al or H_3_BO_3_, to B [41]. The process can be summarized by the following chemical reactions:

(1) $${MFx}^{\left(x-2n\right)-}+nH_{2}O↔{MO}_{n}+{xF}^{-}+2nH^{+}\text{,}$$

(2) $$H_{3}BO_{3}+4H^{++}4F^{-}↔HBF_{4}+3H_{2}O\text{,}$$

(3) $$Al+6H^{++}6F^{-}↔H_{3}AlF_{6}+3/2H_{2}$$

The primary reaction is the hydrolytic-equilibrium reaction (ligand-exchange reaction) of the metal-fluoro complex (MFx^(x−2n)-^) in an aqueous solution (Equation (1)). By adding H_3_BO_3_ or Al metal, which reacts easily with F^−^ ions in this reaction system, a more stable complex is formed in the reactions shown in Equations (2), (3), thereby consuming free F^−^ ions. The equilibrium in Equation (1) shifts toward metal-oxide deposition according to the mass-action law. This naturally causes the reaction in Equation (1) to proceed, resulting in the formation of an oxide thin film on the immersed substrate. Various 2D materials and TiO_2_ hybrids, such as graphene, vanadium oxide, and iron oxide, have been prepared using the LPD method [15]. Herein, we demonstrate the possibility of etching and exfoliating Ti_3_AlC_2_ in the liquid phase in the presence of (NH_4_)_2_TiF_6_. We further show that the TiO_2_, which precipitated using the (NH_4_)_2_TiF_6_ liquid-phase volumetric method, volumetrically formed in the Ti_3_C_2_ MXene layer simultaneously with etching.

This is the first report on the surface microstructure and photocatalytic activity of Ti_3_C_2_ MXene and TiO_2_ hybrids obtained using the LPD method. This work will facilitate the synthesis and development of hybrid materials and the applications of 2D MXene materials and TiO_2_. Owing to the synergistic effect of the slit-like structures of MXene and TiO_2_, MXene is expected to be a promising material for photoresponsive sensors.

## 2 Experimental details

### 2.1 Materials

Ti_3_AlC_2_ (MAX Phase 312, Ti_3_AlC_2_, powder particles ≤200 μm) was provided by Sigma-Aldrich. (NH_4_)_2_TiF_6_ (MW: 197.93), methylene blue (MB), and ethanol were purchased from Fujifilm Wako Pure Chemical Co. H_3_BO_3_ was purchased from Kanto Chemical Co., Inc. All chemicals used in the present study were reagent-grade. Anatase TiO_2_ nanoparticles (<25 nm) were provided by Sigma-Aldrich.

### 2.2 Preparation of the Ti_3_C_2_/TiO_2_ hybrids

First, (NH_4_)_2_TiF_6_ in 80 mL of deionized water was transferred to a stirring plate and stirred for 5 min at 25 °C to prepare a 1.0 M (NH_4_)_2_TiF_6_ solution. The Ti_3_AlC_2_ MAX phase powder (800 mg) was then added to the (NH_4_)_2_TiF_6_–water mixture to prepare a 10 mg/mL MAX phase suspension. The reaction mixture was stirred at 500 rpm for 24 h at 60 °C. The mixed solution was then centrifuged at 4000 rpm for 20 min, the supernatant was discarded, and the residue was collected. The residue was washed three times with distilled water and ethanol. Then, the mixture was filtered through a polyvinylidene difluoride membrane (pore size, 0.65 μm) to collect Ti_3_C_2_/NH_4_TiOF_3_, which was further dried in an oven at 60 °C for 12 h. The dried Ti_3_C_2_/NH_4_TiOF_3_ sample (0.139 g) was placed in a newly prepared 40 mL H_3_BO_3_ solution (0.5 mol L^−1^), stirred for 5 min, and the suspension was maintained at 60 °C for 3 h. The white-colored product was then separated by centrifugation, washed three times with deionized water, and dried at 60 °C for 12 h.

### 2.3 Characterization

The phase constituents of the synthesized products were analyzed by X-ray diffraction (XRD; Rigaku SmartLab, Japan) using a powder diffractometer with Cu Kα radiation in the Bragg–Brentano configuration at 40 kV and 40 mA for angles in the 5–80° range. High-resolution field-emission scanning electron microscopy (FE-SEM) and energy-dispersive X-ray spectroscopy (EDX; JSM-7500 F, JEOL, Japan) were used to observe the nanostructures and surface properties of the synthesized products. Transmission electron microscopy (TEM) was performed using a JEOL JEM 2100 Plus field-emission TEM. The ultraviolet–visible (UV–Vis) spectra of the samples were recorded using a V-550 instrument (JASCO, Japan). The thermogravimetry (TG) and differential thermal analysis (DTA) measurements were performed using a thermogravimetric/differential analyzer (TG 8120; Thermo Plus, Rigaku, Corp., Tokyo, Japan) in a nitrogen atmosphere at a gas flow rate of 100 mL/min and a heating rate of 20 °C/min within the temperature range from 25 to 1000 °C. Raman spectroscopy was performed using a RENISHAW inVia Reflex Raman spectrometer (excitation wavelength, 532 nm). X-ray photoelectron spectroscopy (XPS) was performed using a Thermo Scientific ESCALAB 250Xi XPS microprobe.

### 2.4 Photocatalytic tests

Photocatalytic activity was evaluated by decomposing MB under UV irradiation. The photocatalyst (30 mg) was added to 30 mL of distilled water containing MB (10 mg/L). Before UV irradiation, the suspension was stirred in the dark for 120 min to ensure adsorption-desorption equilibrium. The suspension was then irradiated using a UV lamp (23 W, PU-21, TOPCON) at 365 nm for 120 min with magnetic stirring.

## 3 Results and discussion

**Scheme 1:**
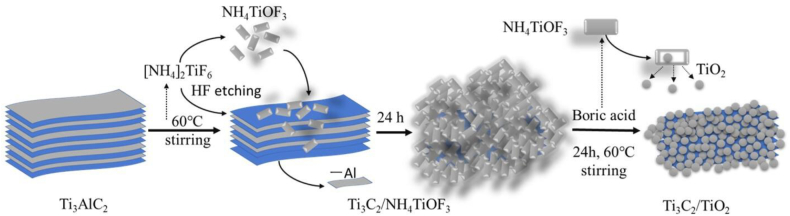
Schematic of the Ti_3_AlC_2_ exfoliation process and the subsequent formation of Ti_3_C_2_/TiO_2_ hybrids.

The synthesis of the Ti_3_C_2_/TiO_2_ hybrids, derived from Ti_3_AlC_2_ and (NH_4_)_2_TiF_6_, is shown in Scheme 1. First, NH_4_TiOF_3_ microcrystals and HF are formed by adding (NH_4_)_2_TiF_6_ in excess to a bulk solution of Ti_3_AlC_2_. The HF acid-free solution selectively etches the Al atoms of Ti_3_AlC_2_ and easily gets consumed during this process, which promotes the mild hydrolysis of (NH_4_)_2_TiF_6_ to form NH_4_TiOF_3_ crystals and HF acid. After stirring and reacting in the mixed solution at 60 °C for 24 h, Ti_3_C_2_/NH_4_TiOF_3_ is formed, in which Ti_3_C_2_ MXene nanosheets are wrapped with NH_4_TiOF_3_ crystals without exfoliation. Next, the obtained Ti_3_C_2_/NH_4_TiOF_3_ is added to a 0.5 M H_3_BO_3_ solution at 60 °C for 24 h under constant stirring. In this reaction, H_3_BO_3_ acts as a fluoride scavenger, thereby consuming excess F^−^ ions and eluting ammonium and fluoride ions from NH_4_TiOF_3_ crystals, which then decays to anatase-type TiO_2_.

Scheme 1 The formation reactions of the Ti3C2/TiO2 hybrids are as follows.

(4) $${\left({NH}_{4}\right)}_{2}TiF_{6}+H_{2}O↔{NH}_{4}{TiOF}_{3}+2HF+{NH}_{4}F$$

(5) $${Ti}_{3}{AlC}_{2}+3HF↔{Ti}_{3}C_{2}+{AlF}_{3}+\left(3/2\right)H_{2}\text{,}$$

(6) $$4{NH}_{4}{TiOF}_{3}+{3H}_{3}{BO}_{3}↔{4TiO}_{2}+{4NH}_{4}^{+}+{3BF}_{4}^{-}+{OH}^{-}+{4H}_{2}O$$

When (NH_4_)_2_TiF_6_ is immersed in a bulk solution of Ti_3_AlC_2_, the following reactions occur: First, a reaction between HF and Ti_3_AlC_2_ may take place (Equation (5)) [31]. The NH_4_TiOF_3_ crystals and HF form by the reaction of (NH_4_)_2_TiF_6_ with H_2_O (Equation (4)), whereas the hydrolysis reaction of NH_4_TiOF_3_ crystals and H_3_BO_3_ takes place as described in Equation (6) [42]. The (NH_4_)_2_TiF_6_ ionizes into TiF_6_^2−^ and NH_4_^+^ ions after its complete dissolution in water. Six F atoms are coordinated to Ti in TiF_6_^2−^ ions, and when the F^−^ are sequentially replaced by hydroxide ions (OH^−^), the reaction proceeds as described, yielding [TiF_3_(OH)_3_]^2-^. The [TiF_3_(OH)_3_]^2-^ ions formed by this ligand-exchange reaction further react with NH_4_^+^ ions to form NH_4_TiOF_3_ crystals. In conventional LPD, H_3_BO_3_ acts as a fluoride scavenger and consumes excess F^−^ ions.

In this study, the hydrolysis of [TiF_6_]^2-^ could not be completed in the presence of Ti_3_AlC_2_, and a stable intermediate product, NH_4_TiOF_3_, was obtained. H_3_BO_3_ played a critical role in scavenging the F^−^ ions generated during the hydrolysis of (NH_4_)_2_TiF_6_. By reacting with HF, H_3_BO_3_ assisted in maintaining a small amount of HF in the reaction solution, thereby promoting the leftward progress of the reaction. Similar to HF, H_3_BO_3_ consumed F^−^ ions in solution and produced stable BF^4−^ ions. Huang et al. investigated the effect of H_3_BO_3_ concentration on the LPD process and reported that higher concentrations of H_3_BO_3_ suppressed HF in solution, resulting in the formation of larger TiO_2_ particles [41]. This effect was attributed to the hindered hydrolysis of [TiF_6_]^2-^ ions. In addition, the reaction rate was significantly slow when the concentration of [TiF_6_]^2-^ ions was below 0.05 mol L^−1^, resulting in longer precipitation times due to supersaturation. This could be due to the slow rate at which Ti_3_AlC_2_ consumed HF acid, which inhibited the hydrolysis of Ti(IV) ions. Ti_3_AlC_2_ served as a surfactant similar to that used for the synthesis of the conventional NH_4_TiOF_3_ crystals. In the presence of water and oxygen, MXene oxidized at a very fast rate, transforming its surface to TiO_2_ with time constants of approximately 5 days for Ti_3_C_2_T_x_ and 7 h for Ti_2_CT_x_ [43]. TiF_6_^2−^ ions combined with Ti–OH on the Ti_3_C_2_ MXene surface, and some F^−^ ions were replaced by OH and grew into Ti_3_C_2_ MXene.

**Fig. 1:**
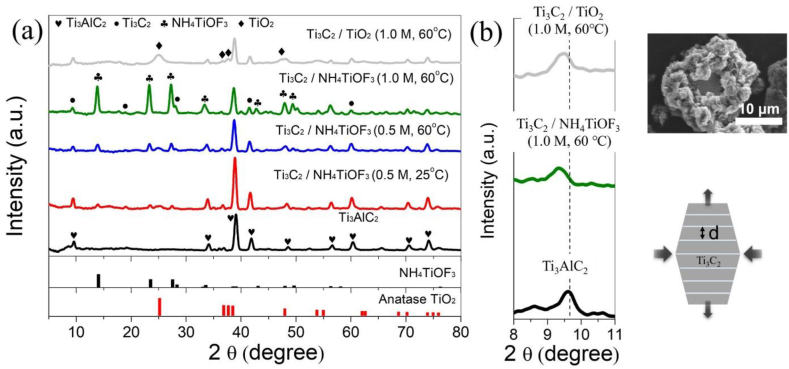
(A) X-ray diffraction pattern (XRD) of Ti_3_AlC_2_, Ti_3_C_2_/NH_4_TiOF_3_ (0.5 M, 25 °C), Ti_3_C_2_/NH_4_TiOF_3_ (0.5 M, 60 °C), Ti_3_C_2_/NH_4_TiOF_3_ obtained by reacting 1.0 M (NH_4_)_2_TiF_6_ at 60 °C, and Ti_3_C_2_/TiO_2_ hybrids. (TiO_2_ from JCPDS No. 21–1272; NH_4_TiOF_3_ from JCPDS No. 54–0239.) (b) Zoom-ins into the 8–11° 2θ range, showing (002) peaks. A diagram of the expansion and contraction of Ti_3_C_2_/NH_4_TiOF_3_ is shown as well.

The XRD patterns of the Ti_3_C_2_/TiO_2_ hybrids are shown in Fig. 1. The characteristic peaks of the Ti_3_C_2_ MXene phase and NH_4_TiOF_3_ crystals are observed at high etchant concentrations when the synthesis is performed at 60 °C for 24 h. Compared with the lower synthesis temperature of 25 °C, etching of Ti_3_AlC_2_ with (NH_4_)_2_TiF_6_ at 60 °C clearly attenuates the 9.6° reflection of Ti_3_AlC_2_ and induces a shift toward the smaller angle of 9.3°. The Al layer is removed from Ti_3_AlC_2_, confirming the formation of the Ti_3_C_2_ MXene phase [44]. The Ti_3_AlC_2_ peaks at (002) at 9.6° and (005) at 14° shift slightly toward smaller angles; hence, the overlapping multilayer structure is retained when the Ti_3_C_2_ MXene becomes a single layer during coating. The peak intensities of Ti_3_AlC_2_ at 34.2°, 41.9°, 60.4°, and 71.1° decreased. Other typical Ti_3_C_2_ MXene peaks, such as (006) at 18.9°, (008) at 28.1°, (0012) at 41.5°, and (110) at 60.0°, are observed, indicating that Ti_3_C_2_ MXene is formed by the removal of the Al interlayer. After etching with (NH_4_)_2_TiF_6_, a sharp peak is observed at 2θ = 39°, corresponding to the (104) plane of Ti_3_AlC_2_. This indicates that the outer Al layer of Ti_3_AlC_2_ is selectively etched; however, Al may remain in the center. The largest particle size (≤200 μm) of any commercially available MAX phase precursor was used in this study. Therefore, long etching times and high temperatures may be required to penetrate the central Al layer. This allows the formation of a planar heterojunction between the Ti_3_C_2_ MXene and Ti_3_AlC_2_.

Typically, NH_4_TiOF_3_ crystals are synthesized from solutions containing (NH_4_)_2_TiF_6_ and H_3_BO_3_ in the presence of nonionic surfactants at low temperatures [45]. Alternatively, if the molar ratio of (NH_4_)_2_TiF_6_ to H_3_BO_3_ is approximated, NH_4_TiOF_3_ mesocrystals can be obtained under surfactant-free conditions [46]. Here, typical peaks are observed at 23.4°, 33.8°, 48.1°, and 56.3° after the heating reaction with 1.0 M of (NH_4_)_2_TiF_6_ at 60 °C (hereafter abbreviated as Ti_3_C_2_/TiO_2_ hybrids) and are indexed to the (101) crystal planes of the synthesized NH_4_TiOF_3_ crystals [47]. The Ti_3_C_2_/NH_4_TiOF_3_ product before treatment with H_3_BO_3_ is consistent with well-crystallized pure good-phase NH_4_TiOF_3_ (JCPDS No. 54–0239). The 25° reflection, indexed to the (101) reflection of anatase TiO_2_ (JCPDS No. 21–1272), is confirmed based on the XRD pattern of the Ti_3_C_2_/NH_4_TiOF_3_ product after post-treatment with H_3_BO_3_ [45]. Weak diffraction intensity is observed at 25 °C for the synthesized NH_4_TiOF_3_ crystals. These results indicate that the coverage of the synthesized NH_4_TiOF_3_ crystals on the Ti_3_C_2_ layer is sparse when the concentration of the (NH_4_)_2_TiF_6_ agent and the reaction temperature are low.

Intercalation with H_2_O, NH_3_, dimethyl sulfoxide (DMSO), and urea is thought to increase the d-spacing of Ti_3_C_2_ MXene and further promote the exfoliation of MXene 2D sheets [48,49]. As shown in Fig. 1(b), the synthesized NH_4_TiOF_3_ crystals grow through the gaps in the transverse layers of Ti_3_C_2_ MXene, possibly causing Ti_3_C_2_ MXene to expand longitudinally. The (002) peak of Ti_3_AlC_2_ is located at 9.6° (d-spacing, 9.2 Å), while the (002) peak of the hybrids synthesized for 1.0 M at 60 °C shifts to 9.3° (d-spacing, 9.5 Å). The results indicate that the synthesized NH_4_TiOF_3_ crystals are volumetrically loaded from the horizontal direction of the Ti_3_C_2_ MXene layer, and the applied force increases the interlayer spacing and compromises the original crystallinity of Ti_3_C_2_ MXene [49]. As the compositing progresses, the (002) peak corresponding to the broadening of the interlayer distance of Ti_3_C_2_ MXene shifts toward smaller angles. The conversion of NH_4_TiOF_3_ crystals to TiO_2_ nanoparticles with H_3_BO_3_ results in the collapse of the NH_4_TiOF_3_ crystal structure and a shift of the (002) peak toward the wide angle of 9.5° (d-spacing, 9.3 Å). These results suggest that the Ti_3_C_2_/TiO_2_ hybrids may exhibit flexibility and elasticity in response to the entry and exit of small molecules.

The crystallite sizes of the Ti_3_AlC_2_, Ti_3_C_2_/NH_4_TiOF_3_, and Ti_3_C_2_/TiO_2_ hybrids were calculated using the Debye-Scherer equation [50]:

(7) $$D=\frac{K\lambda }{\beta_{hkl}\;\mathrm{Cos}\;\theta }$$

where D is the crystallite size, K is the shape factor, λ is the X-ray wavelength of the Cu source used in XRD (1.5406 Å); Bragg's angle is given by θ; and β represents the full width at half maximum (FWHM).

With respect to the Ti_3_C_2_ (002) planes for all samples, the microstrain was analyzed using the simplified Williamson-Hall (W–H) method [51].

(8) $$\beta_{hkl}\;\mathrm{Cos}\;\theta =\frac{K\lambda }{D}+4\varepsilon \;\sin\;\theta$$

Table 1 lists the crystallite size and microstrain characteristics of Ti_3_AlC_2_, Ti_3_C_2_/NH_4_TiOF_3_, and Ti_3_C_2_/TiO_2_ hybrids with respect to the Ti_3_C_2_ (002) planes. Ti_3_AlC_2_ exhibits a crystallite size of 13.66 nm, whereas the crystallite sizes of Ti_3_C_2_/NH_4_TiOF_3_ and Ti_3_C_2_/TiO_2_ hybrids are 16.66 and 14.12 nm, respectively. Furthermore, the microstrain of Ti_3_AlC_2_ is 29.97, whereas the Ti_3_C_2_/NH_4_TiOF_3_ and Ti_3_C_2_/TiO_2_ hybrids exhibit lower microstrain values of 26.95 and 27.07, respectively. These results indicate the successful incorporation of TiO_2_ into hybrid materials [52]. A consistent increase in crystallite size was observed along with a decrease in microstrain, suggesting an expansion of the d-spacing upon the introduction of TiO_2_ into Ti_3_C_2_ [53]. The crystallite size of NH_4_TiOF_3_, determined using the (002) plane, is 13.49 nm. Treatment with H_3_BO_3_ dissolves the NH_4_TiOF_3_ nanoparticles, leading to the formation of highly crystalline anatase TiO_2_ with a crystallite size of 4.97 nm in the (101) planes [45].

**Table 1:** The peak position, d-spacing, full width at half maximum, crystallite size, and microstrain analysis obtained from the X-ray diffraction patterns, and particle size analysis from scanning electron microscopic images.

Sample	Peak position (°)	D-spacing (nm)	FWHM	Crystallite size (nm)	Microstrain (*ε*) × 10^3^
Ti_3_AlC_2_	9.61	0.92	0.577	13.66	29.97
Ti_3_C_2_/NH_4_TiOF_3_	9.32	0.95	0.503	16.66	26.95
	13.86		0.481	13.49	17.30
Ti_3_C_2_/TiO_2_ hybrids	9.47	0.93	0.514	14.12	27.07
	25.07		1.847	4.97	36.25

**Fig. 2:**
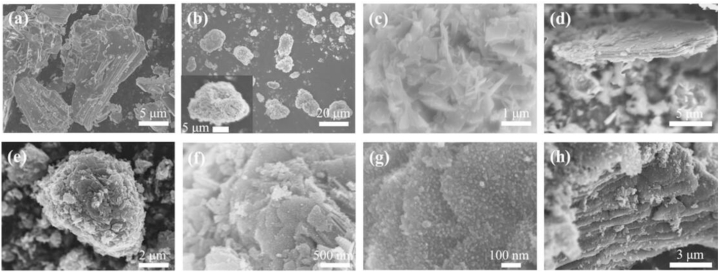
Scanning electron microscopy (SEM) images of (a) Ti_3_AlC_2_, (b–d) Ti_3_C_2_/NH_4_TiOF_3_ (1.0 M, 60 °C), and (e–h) Ti_3_C_2_/TiO_2_ hybrids.

To further explore structural changes in the samples, their morphological evolution was investigated. Fig. 2 demonstrates the FE-SEM images of Ti_3_AlC_2_, Ti_3_C_2_/NH_4_TiOF_3_, and Ti_3_C_2_/TiO_2_ hybrids. Fig. 2(a) shows the layered structure of Ti_3_AlC_2_. According to Fig. 2(b and c), Ti_3_C_2_ MXene is uniformly covered with NH_4_TiOF_3_ crystals (thickness: 50 nm; approximate length: 250 nm) after reacting with 1.0 M (NH_4_)_2_TiF_6_ at 60 °C for 24 h. The Ti_3_C_2_/NH_4_TiOF_3_ coverage is uniform, and, as shown in Fig. 2(d), etching with (NH_4_)_2_TiF_6_ eliminates the Al layer of Ti_3_AlC_2_, resulting in the formation of an accordion-like structure. NH_4_TiOF_3_ crystals are not observed even after reacting for 12 h. More uniform complexes are observed at higher concentrations of (NH_4_)_2_TiF_6_ than those at lower concentrations; therefore, 1.0 M of (NH_4_)_2_TiF_6_ is determined as the optimal condition, compared with 0.25 M and 0.5 M. Furthermore, NH_4_TiOF_3_ crystals are not observed after 12 h of reaction. As shown in Fig. 2(e and f), when Ti_3_C_2_/NH_4_TiOF_3_ reacts with H_3_BO_3_, a uniform hybrid structure is maintained, whereas the NH_4_TiOF_3_ crystals collapse. The high-magnification FE-SEM image in Fig. 2(g) shows the formation of TiO_2_ nanoparticles on the hybrid surface. Fig. 2(h) shows that the formed TiO_2_ particles sufficiently wrap the Ti_3_C_2_ MXene surface and are evenly distributed on the edges and surface of the Ti_3_C_2_ MXene sheet. A striking similarity is observed between the anatase structure and the structure inferred for NH_4_TiOF_3_ [45]. The position of the [001] plane of the Ti atom is similar in both; however, the plane is separated by ammonium ions in a lamellar structure in the case of NH_4_TiOF_3_. The reaction with H_3_BO_3_ changes the orientation of NH_4_TiOF_3_ to anatase. In addition, the high-magnification SEM images (Fig. 2) demonstrate the presence of NH_4_TiOF_3_ and TiO_2_ nanocrystals. The particle sizes of NH_4_TiOF_3_ and TiO_2_, as observed by SEM, are 33.09 ± 0.97 and 9.9 ± 0.53 nm, respectively. These values are larger than the crystallite sizes determined using the XRD patterns. This is possibly because the particles grow uniformly in a solution and agglomerate during the centrifugation and drying processes.

**Fig. 3:**
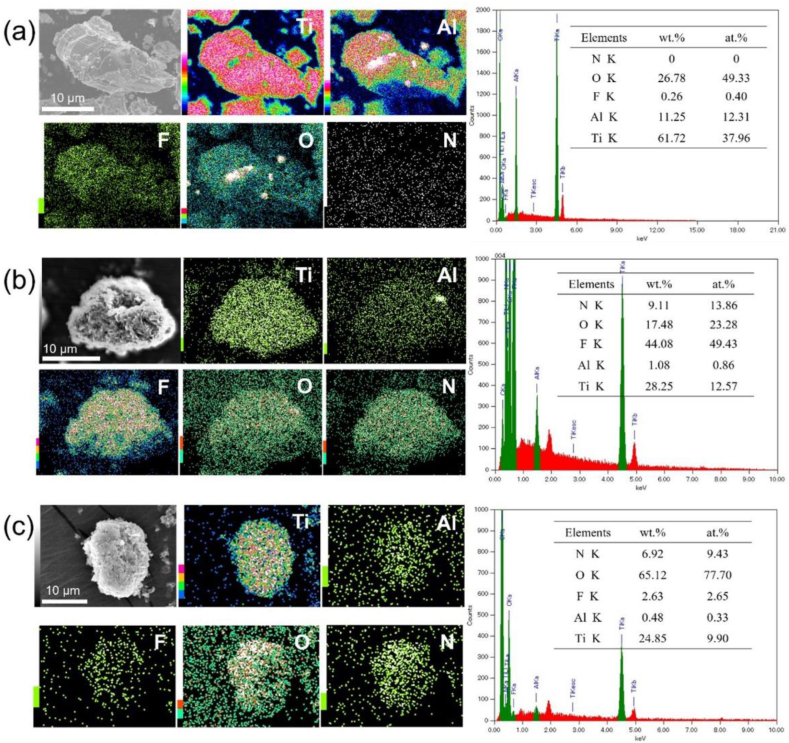
Energy dispersive X-ray spectroscopy (EDX) mapping results for (a) Ti_3_AlC_2,_ (b) Ti_3_C_2_/NH_4_TiOF_3_ (1.0 M, 60 °C) and (c) Ti_3_C_2_/TiO_2_ hybrids of the elemental Ti, Al, F, O, and N EDX spectra.

Comparing the EDX mapping images of Ti_3_C_2_/NH_4_TiOF_3_ (Fig. 3(b)) to those of the Ti_3_AlC_2_ MAX (Fig. 3(a)) reveals the presence of the constituent elements Ti, O, F, and C, as well as Al by-products formed during the exfoliation and de-stacking processes. The homogeneous bonding of the NH_4_TiOF_3_ crystals to Ti_3_C_2_ MXene is clearly observed. As shown in Fig. 3(c), Ti_3_C_2_/TiO_2_ hybrids form after the reaction with H_3_BO_3_, indicating that the amounts of F and Al elements decrease whereas the constituent elements Ti, O, and C remain the same. N remains as a byproduct of NH_4_^+^. Fang et al. successfully obtained anatase TiO_2_ nanobricks with a hierarchical hollow structure by post-treating mesocrystalline NH_4_TiOF_3_ nanobricks with H_3_BO_3_ [54]. Therefore, TEM analysis was performed to reveal the internal structure of the NH_4_TiOF_3_ crystal after its collapse.

**Fig. 4:**
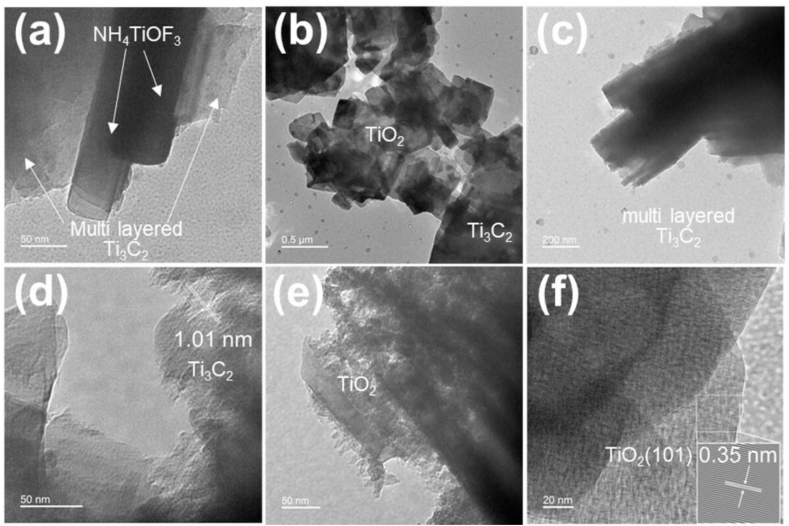
Transmission electron microscopy (TEM) images of (a) Ti_3_C_2_/NH_4_TiOF_3_ (1.0 M, 60 °C) and (b–f) Ti_3_C_2_/TiO_2_ hybrids.

Fig. 4 shows the TEM images of Ti_3_C_2_/NH_4_TiOF_3_ (1.0 M, 60 °C) and Ti_3_C_2_/TiO_2_ hybrids. As shown in Fig. 4(a), Ti_3_C_2_/NH_4_TiOF_3_ exhibits the NH_4_TiOF_3_ crystal structure on Ti_3_C_2_ MXene nanosheets. The NH_4_TiOF_3_ precursor is a nanobrick with edge length of 200–300 nm and thickness of 40–60 nm. In the Ti_3_C_2_/TiO_2_ hybrids obtained by the reaction with H_3_BO_3_ (Fig. 4(b)), the NH_4_TiOF_3_ precursor crystal structure collapses into hollow nanobrick structures. The conversion of the NH_4_TiOF_3_ crystals to TiO_2_ (observed in the TEM images) agrees well with the conclusions from SEM images (Fig. 2(f)) and with the literature findings [54]. As shown in Fig. 4(c and d), the Ti_3_C_2_/TiO_2_ hybrid exhibits microscale bulk particles with a multilayer structure, with HR-TEM revealing a typical multilayer crystal structure with an interlayer spacing of ∼1.01 nm. Fig. 4(e) shows that numerous TiO_2_ nanoparticles are uniformly anchored in the multilayered Ti_3_C_2_ MXene. The interplane distance of the TiO_2_ is 0.35 nm, which exactly matches the primary plane of anatase TiO_2_ (101) (Fig. 4(f)).

**Fig. 5:**
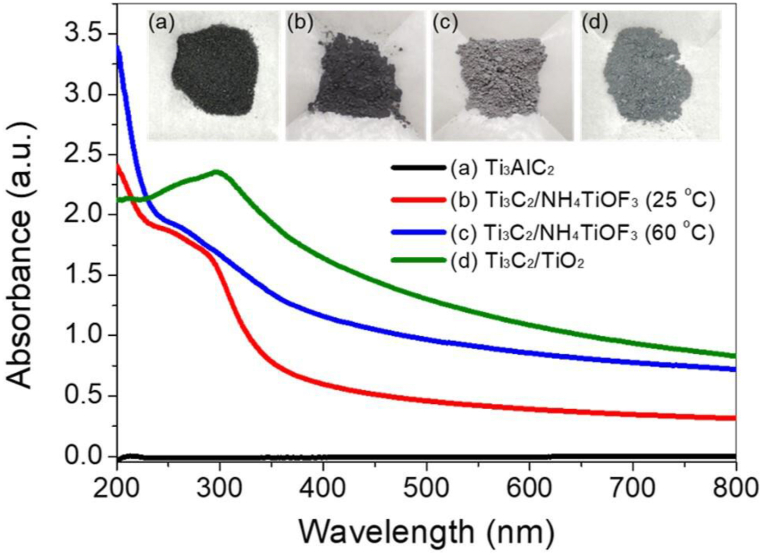
Comparative ultraviolet–visible (UV–Vis) absorbance spectra of Ti_3_AlC_2_, Ti_3_C_2_/NH_4_TiOF_3_ (0.5 M, 25 °C), Ti_3_C_2_/NH_4_TiOF_3_ (1.0 M, 60 °C), and Ti_3_C_2_/TiO_2_ hybrids in aqueous solutions (1.0 mg/mL concentration).

To investigate the optical absorption rate, the UV–Vis spectra of the samples were measured using a UV–Vis spectrophotometer. Aqueous solutions of each sample were prepared at a concentration of 1.0 mg/mL for the UV–Vis measurements. As shown in Fig. 5, Ti_3_C_2_/NH_4_TiOF_3_ exhibits UV and visible absorption spectra owing to the black nature of the hybrids synthesized at 25 °C. The unmodified Ti_3_AlC_2_ MAX phase precipitates rapidly in aqueous solution, making the absorption range unclear. Furthermore, the optical absorption of Ti_3_C_2_/TiO_2_ hybrids exhibits a red shift and increases in the 350–800 nm range, indicating that the intensity of the *C*–TiO_2_ absorption edge increases in the visible light region. This is attributed to the adsorption of carbonaceous material [30]. The good light absorption capacity of Ti_3_C_2_/TiO_2_ hybrids may be advantageous for photocatalysis. The UV absorption spectrum of Ti_3_C_2_/NH_4_TiOF_3_ shows peaks at 260 nm. This absorption is considered to correspond to the band gap energy of the oxidized Ti_3_C_2,_ which was also predicted by theoretical calculations in the literature [55]. When Ti_3_C_2_/NH_4_TiOF_3_ reacts with H_3_BO_3_, the UV absorption in the 200–230 nm range is weakened, indicating that the absorption originates from the NH_4_TiOF_3_ crystal.

The Ti_3_C_2_/NH_4_TiOF_3_ (0.5 M, 25 °C) exhibits no significant changes in the color tone and shape of the product, whereas the Ti_3_C_2_/NH_4_TiOF_3_ (1.0 M, 60 °C) and Ti_3_C_2_/TiO_2_ hybrid products synthesized at 60 °C become fine particles and their color tone changes from black to gray (Fig. 5), suggesting that the Ti_3_C_2_ MXene and NH_4_TiOF_3_ precursors are successfully combined.

**Fig. 6:**
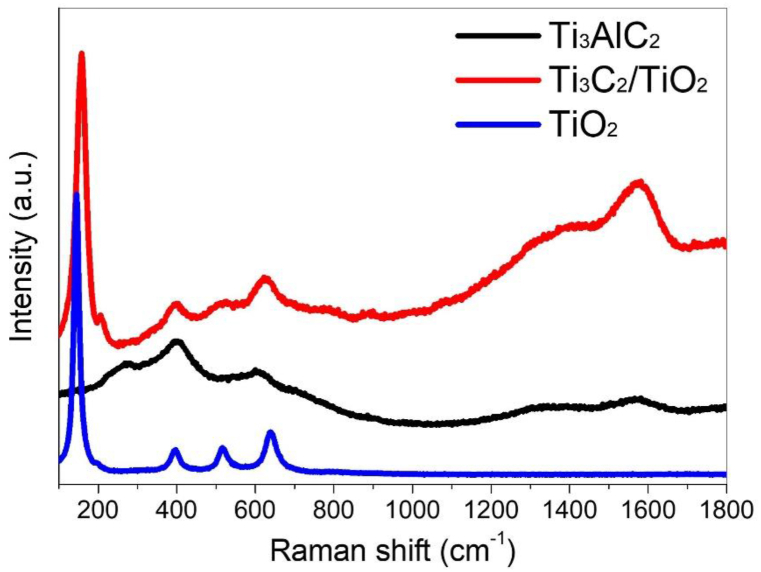
Raman spectra of Ti_3_AlC_2_, Ti_3_C_2_/TiO_2_ hybrids, and pure TiO_2_ anatase nanoparticles.

Fig. 6 shows the Raman spectra of Ti_3_AlC_2_ and Ti_3_C_2_/TiO_2_ hybrids. The peaks at 395 and 610 cm^−1^ in the spectrum of Ti_3_AlC_2_ agree well with the main features of Ti_3_AlC_2_, which are caused by the shear and longitudinal vibrations of Ti and Al atoms [56]. The labeled peaks in the spectrum of the Ti_3_C_2_/TiO_2_ hybrid, at 157 (E_g_), 395 (B_1g_), 505 (A_1g_ + B_1g_), and 624 (E_g_) cm^−1^ are characteristic optical modes of anatase TiO_2_ [57]. These peaks are detected at 144, 396, 516, and 638 cm^−1^ for commercially available anatase TiO_2_ nanoparticles. However, compared to pure anatase TiO_2_ nanoparticles, the frequency of the strongest Eg mode, which is attributed to the external vibration of the Ti–O bond, is significantly blue-shifted to 157 cm^−1^ in Ti_3_C_2_/TiO_2_ hybrids, accompanied by an increase in peak width, thereby indicating that the crystal defects in TiO_2_ of Ti_3_C_2_/TiO_2_ hybrids are increased. These crystal defects form in the contact region between Ti_3_C_2_ and TiO_2_, affecting the vibrational frequency of anatase TiO_2_ and acting as a trap for the photoelectrons produced. The high carrier mobility of Ti_3_C_2_ results in the rapid transfer of excited electrons to the Ti_3_C_2_ thin film structure, providing a reaction site for one-electron reduction of oxygen.

Furthermore, after the TiO_2_ coating treatment, a shift in the Raman band centered at 610 cm^−1^ toward higher wavelengths (624 cm^−1^) is observed, possibly owing to the increased interlayer distance [58]. The peaks in the spectrum of the Ti_3_C_2_/TiO_2_ hybrid are longer and wider than those in the Ti_3_AlC_2_ spectrum, which is attributed to the intercalation of surface groups by etching, as confirmed by the XRD spectra. The peak at 505 cm^−1^ is attributed to the anatase phase of TiO_2_. The two peaks in the 1200–1700 cm^−1^ range are attributed to the D band at 1401 cm^−1^ and the G band at 1585 cm^−1^ of graphitic carbon, indicating the presence of carbon and disorder in the sample. The G band is due to the in-plane vibrations of the C atoms in the SP2 bonds, whereas the D band is due to the out-of-plane vibrations caused by structural defects [59]. The D/G ratio for the Ti_3_C_2_/TiO_2_ hybrid is 0.86, indicating that the graphitic carbon formed by etching TiO_2_ is homogeneous, regardless of the formation temperature. This indicates that the growth of TiO_2_ likely disrupts the structure of the Ti_3_C_2_ MXene sheet, causing the innermost Ti atoms in the Ti_3_AlC_2_ structure to move outward and react with oxygen, leaving C atoms on the surface. These facts are consistent with the conclusions of various studies, suggesting that Ti_3_C_2_/TiO_2_ hybrids have been successfully synthesized.

**Fig. 7:**
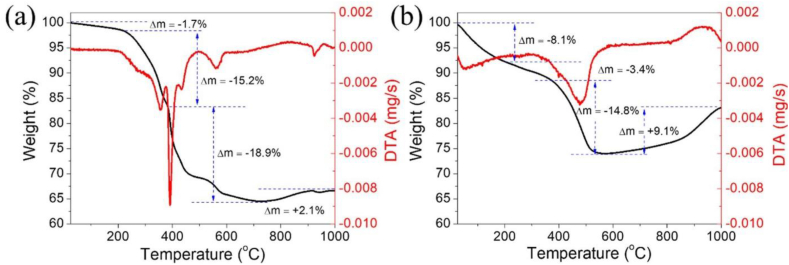
The thermogravimetry (TG) and differential thermal analysis (DTA) curves of (a) Ti_3_C_2_/NH_4_TiOF_3_ (1.0 M, 60 °C) and (b) Ti_3_C_2_/TiO_2_ hybrids in a nitrogen atmosphere.

Fig. 7(a) shows the TG-DTA curves of the as-prepared Ti_3_C_2_/NH_4_TiOF_3_ (1.0 M, 60 °C). Fig. 7(b) shows the curves for Ti_3_C_2_/TiO_2_ hybrids in the temperature range of 25–1000 °C in a nitrogen atmosphere. The TG plots are divided into four regimes. In the first regime of weight loss (temperatures in the 25–210 °C range), a mass loss of approximately 1.7% occurred for the Ti_3_C_2_/NH_4_TiOF_3_ (1.0 M, 60 °C) and 8.1% for the Ti_3_C_2_/TiO_2_ hybrid sample. This is attributed to the removal of physically and chemically entrapped water. Sharp endothermic peaks in the TG curves at 250–375 °C for Ti_3_C_2_/NH_4_TiOF_3_ (1.0 M, 60 °C) and at 220–370 °C for Ti_3_C_2_/TiO_2_ hybrids, corresponding to weight losses of 15.2% and 3.4% for Ti_3_C_2_/NH_4_TiOF_3_ (1.0 M, 60 °C) and Ti_3_C_2_/TiO_2_ hybrids, respectively, are observed in the second regime. This is attributed to the decomposition of crystallization water and organic compounds from the OH groups on the Ti_3_C_2_/NH_4_TiOF_3_ and Ti_3_C_2_/TiO_2_ hybrids. The third weight-loss regime is due to the crystallization of TiO_2_. The weight of Ti_3_C_2_/TiO_2_ hybrid decreases by 14.8% at 370–580 °C, which is associated with the transformation of Ti from anatase to rutile phase [60]. The weight of Ti_3_C_2_/NH_4_TiOF_3_ (1.0 M, 60 °C) decreases by 18.9% in the temperature range of 375–730 °C, which is associated with the transformation of Ti from the NH_4_TiOF_3_ precursor to the anatase and rutile phases. The DTA curve confirms that TiO_2_ crystallization proceeds faster in the Ti_3_C_2_/TiO_2_ hybrids. The phase transition from anatase to rutile is clearly enhanced in the Ti_3_C_2_/TiO_2_ hybrids. At high temperatures (above 565 °C), the weights of Ti_3_C_2_/TiO_2_ hybrids increase gradually owing to the selective oxidation of Ti_3_C_2_/NH_4_TiOF_3_ when heated with N_2_, which has a very low oxygen content [61]. At such high temperatures, the Ti_3_C_2_/TiO_2_ hybrids react with the previously released oxygen to form oxides [62]. The Ti_3_C_2_/TiO_2_ hybrids exhibit better oxidation resistance than TiC because Al intercalates into TiC to form ternary Ti_3_C_2_/TiO_2_ hybrids [63]. In the final weight-increase regime for both samples, an endothermic peak is observed at approximately 960 °C in the DTA curve. This peak is accompanied by the removal of F groups attached to the surface of Ti_3_C_2_/NH_4_TiOF_3_ (1.0 M, 60 °C) and Ti_3_C_2_/TiO_2_ hybrids and the breakdown of the layered structure, resulting in the disproportionation of Ti_3_C_2_ MXene and the formation of TiO_2_ and TiC phases [17].

**Fig. 8:**
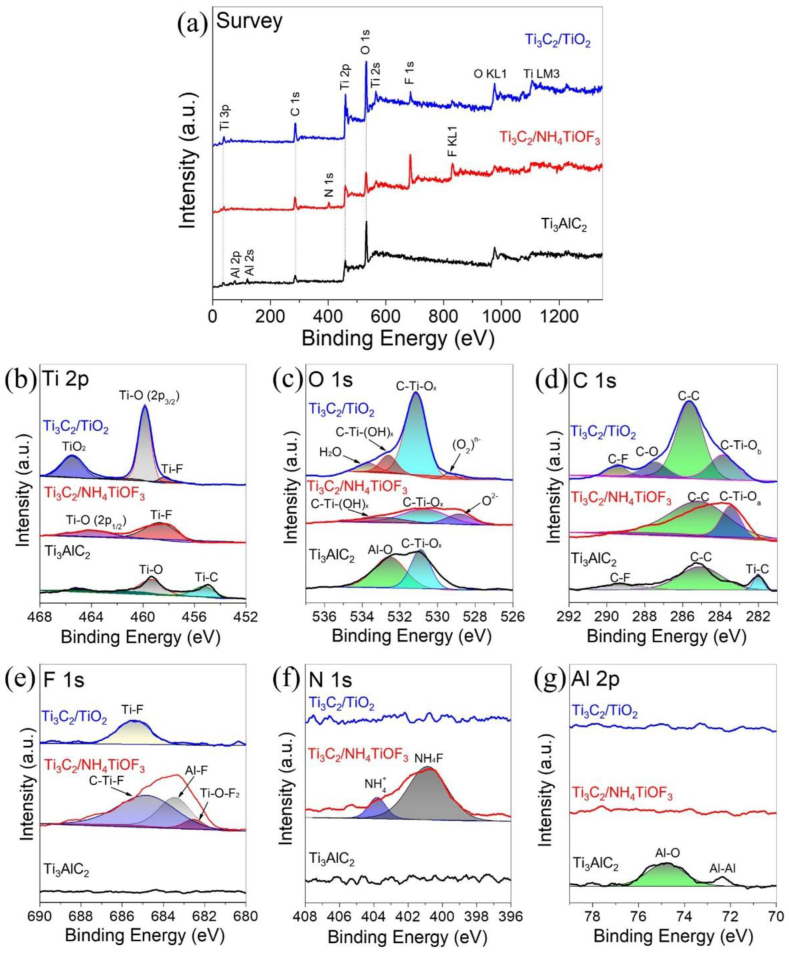
X-ray photoelectron spectra for (a) survey, (b–g) high-resolution spectra of the Ti 2p, O 1s, C 1s, F 1s, N 1s, and Al 2p regions, respectively, of representative samples.

To investigate the formation of TiO_2_ on Ti_3_C_2_, XPS experiments were performed as shown in Fig. 8. Fig. 8(a) shows the XPS survey spectra of Ti_3_C_2_/TiO_2_ hybrids, Ti_3_C_2_/NH_4_TiOF_3_ (1.0 M, 60 °C), and Ti_3_AlC_2_. The Ti_3_C_2_/TiO_2_ hybrids exhibit the peaks of Ti 3p, C 1s, Ti 2p, O 1s, and F1s, thereby indicating the presence of TiO_2_. The survey spectra of Ti_3_C_2_/NH_4_TiOF_3_ show the peaks of Ti 3p, C 1s, N 1s, Ti 2p, O 1s, and F 1s, indicating the presence of NH_4_TiOF_3_ precursors. Notably, the Al 2p peak is absent in the Ti_3_C_2_/TiO_2_ hybrids and Ti_3_C_2_/NH_4_TiOF_3_ samples, indicating that Al is etched from Ti_3_AlC_2_ and that Ti_3_C_2_ is homogeneously coated with TiO_2_ (Fig. 8(g)). As shown in Fig. 8(b), the Ti 2p spectrum of Ti_3_C_2_/TiO_2_ hybrids shows two predominant peaks located at 459.9 and 465.5 eV, corresponding to the Ti–O bond and TiO_2_, respectively. For the Ti_3_C_2_/NH_4_TiOF_3_, the two peaks at 458.7 and 464.1 eV can be attributed to the Ti–F bond and 2p1/2 of the Ti–O bond, respectively. Fig. 8(c) shows the O 1s peaks for the Ti_3_C_2_/TiO_2_ hybrids, which exhibit oxidized oxygen (O_2_)^n–^, C–Ti–O_x_, C–Ti–(OH)_x_, and H_2_O peaks with binding energies of 529.4, 531.2, 532.6, and 533.7 eV, respectively. The intensity of the C–Ti–O_x_ peak increases as oxidization progresses because surface and edge Ti–F bonds are converted into Ti–O bonds. The lattice oxygen (O^2−^) peak at 528.8 eV in Ti_3_C_2_/TiO_2_ hybrids is reduced compared to Ti_3_C_2_/NH_4_TiOF_3_, confirming the occurrence of the (O_2_)^n–^ peak due to oxidation of lattice oxygen. As shown in Fig. 8(d), the high-resolution C1s spectrum of Ti_3_C_2_/TiO_2_ hybrids shows C–Ti–O_b_ (283.9 eV), C–C (285.7 eV), C–O (287.5 eV), and C–F (281.51 eV) bonds. On the other hand, the C1s spectrum of Ti_3_C_2_/NH_4_TiOF_3_ shows C–Ti–O_a_ (283.5 eV) and C–C (285.2 eV) bonds. The F 1s and N 1s peaks of Ti_3_C_2_/NH_4_TiOF_3_ are fitted at 682.5, 683.5, 684.8, 400.8, and 403.8 eV, which are assigned to Ti–O–F_2_, Al–F, C–Ti–F, NH_4_F, and NH_4_^+^, respectively, as shown in Fig. 8(e and f).

In this study, a 1.0 M concentration of (NH_4_)_2_TiF_6_ was used to synthesize the precursor, whereas the concentration of H_3_BO_3_ was set at 0.5 M. A relatively low concentration of H_3_BO_3_ ensured the rapid conversion of the NH_4_TiOF_3_ precursor to TiO_2_ at 60 °C without leaving any residue. This is supported by the XPS results, which show the absence of NH_4_^+^ or NH_4_F derived from NH_4_TiOF_3_ in the final Ti_3_C_2_/TiO_2_ product. Elemental analysis confirmed the absence of B, indicating that the products were carefully cleaned with ethanol and water to remove residual impurities.

The etching of MXene with fluoride salts can result in the formation of byproducts such as AlF_3_ and AlF_3_–3H_2_O. Cockreham et al. conducted a study on the etching of Ti_3_C_2_ MXenes with CoF_2_/CoF_3_ and reported that the ionic strength of the etching solution affected the formation of AlF_3_–3H_2_O impurities [64]. Specifically, stable complexation of AlF_3_ was observed within an ionic strength range of approximately 8.5–10 M (Equation (9)), resulting in the presence of AlF_3_–3H_2_O impurities in the Ti_3_C_2_ MXene phase, as confirmed by XRD and SEM analysis.

(9) $$I=\frac{1}{2}\sum_{i=1}^{n}c_{i}z_{i}^{2}$$

where *I* is the molar ionic strength, c_i_ is the molar ionic concentration, and z_i_ is the charge number of the ion. In the current study, the etching solution consisted of (NH_4_)_2_TiF_6_ at a concentration of 1.0 M and an ionic strength of 10.5 M for the MAX phase. The ionic strength exceeds 10 M, indicating that the deposition of AlF_3_ impurities is unlikely. Furthermore, the absence of AlF_3_ precipitates was confirmed by the XRD, XPS, and SEM analyses of the synthesized products. Therefore, the absence of AlF_3_ in the samples used for photocatalytic experiments did not affect the amount of TiO_2_.

Previous studies have used an NH_4_HF_2_ etchant concentration of 1.0 M [38,39]. Furthermore, the etchant concentration of 1.0 M was found to be optimal compared to 0.25 or 0.5 M in this study. The rationale for this choice was based on several key factors. First, a higher concentration of (NH_4_)_2_TiF_6_, the precursor for Ti_3_C_2_ synthesis, resulted in more uniform complex formation than a lower concentration of etchant. A highly homogeneous complex, which was crucial for the subsequent formation of Ti_3_C_2_, was achieved using 1.0 M (NH_4_)_2_TiF_6_. Second, the ionic strengths of both the MAX phase and (NH_4_)_2_TiF_6_ should be considered during synthesis. The ionic strength of 10.5 M played a role in suppressing the formation of unwanted byproducts, such as AlF_3_. The ionic strength could be effectively controlled by maintaining a higher etchant concentration, thereby minimizing the formation of unwanted compounds. Considering these factors, the amount of TiO_2_ present in the composite synthesized with 1.0 M (NH_4_)_2_TiF_6_ was considered optimal for this study. This provided the necessary conditions for the formation of uniform complexes while preventing the formation of unwanted byproducts.

**Fig. 9:**
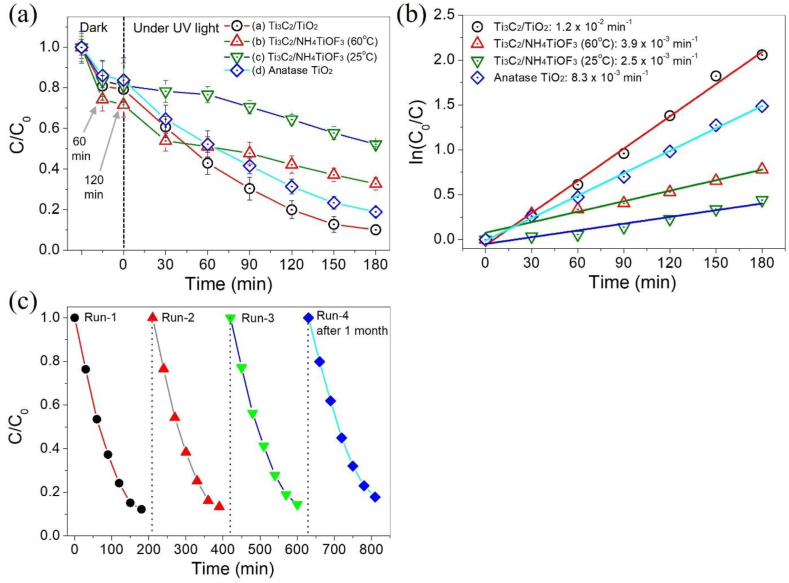
(A) Irradiation time vs. degradation (C/C_0_) curves and (b) the pseudo-first-order kinetics fitting for the photocatalytic degradation of methylene blue (MB) using the synthesized final product, two precursors, and pure TiO_2_ anatase nanoparticles as photocatalysts. (c) Photocatalytic recyclability and stability of the Ti_3_C_2_/TiO_2_ hybrids in the photodegradation of MB. (For interpretation of the references to color in this figure legend, the reader is referred to the Web version of this article.)

Fig. 9(a) shows the photocatalytic degradation capacity of the final product and two precursors and commercially available anatase TiO_2_ nanoparticles. The adsorption of organic molecules onto TiO_2_ surfaces involves physical van der Waals forces, electrostatic interactions, and chemical bonding with acidic sites and hydroxyl groups [65]. Wiedmer et al. reported that six commercial TiO_2_ species showed the strongest degradation to MB at neutral pH 6.7 out of pH 3, 6.7, and 9 [66]. In neutral aqueous solution, MB is oxidized, which favors the adsorption on the catalyst surface when the catalyst forms oxygen-centered radicals (•OH, O_2_^−•^/•OOH). MB is fully positively charged at a pH > 5, and the mole fraction of positively charged MB is approximately 15% and 55% at pH 3 and 4, respectively [67]. These observations demonstrate the strong dependence of the protonation and deprotonation of MB on the pH of the solution. Similarly, the surface charge of anatase TiO_2_ was reported to remain neutral in the pH range of 6–8 [68]. By conducting the study under neutral conditions, the authors aimed to eliminate the potential complexities arising from excessive protonation or deprotonation, thereby ensuring an accurate assessment of the photodegradation of MB with TiO_2_.

The amounts of MB adsorbed on Ti_3_C_2_/TiO_2_ hybrids, Ti_3_C_2_/NH_4_TiOF_3_ (60 °C), Ti_3_C_2_/NH_4_TiOF_3_ (25 °C), and anatase TiO_2_ nanoparticles are 0.22 ± 0.0087, 0.29 ± 0.015, 0.20 ± 0.014, and 0.17 ± 0.013 mmol g^−1^, respectively, when kept in the dark for 120 min. After 180 min of UV light illumination, the degradation rates of Ti_3_C_2_/TiO_2_ hybrids, Ti_3_C_2_/NH_4_TiOF_3_ (60 °C), Ti_3_C_2_/NH_4_TiOF_3_ (25 °C), and commercially available anatase TiO_2_ nanoparticles are 89.9, 67.2, 47.8, and 81.0%, respectively. The reaction rate of Ti_3_C_2_/NH_4_TiOF_3_ decreases after 30 min of irradiation. The low photocatalytic activity of Ti_3_C_2_/NH_4_TiOF_3_ can be explained by the following aspects. A commonly used UV light source has a peak intensity at 365 nm, which does not ideally match the absorption spectrum of NH_4_TiOF_3_. In addition, NH_4_TiOF_3_ has low electron mobility along certain crystallographic directions because of its layered structure, which may result in inefficient charge transport from the bulk to the active sites on the surface. Furthermore, electrons flow to O_2_ via Ti–F in NH_4_TiOF_3_ crystals to form superoxide anions (O_2_^•–^), which are converted into hydroxyl radicals (OH^•^) via protonation and *O*–O bond cleavage [69]. Thus, the cationic dye MB is preferentially adsorbed onto the surface of Ti_3_C_2_/NH_4_TiOF_3_, which is negatively charged in aqueous solutions via electrostatic interactions. The intensity of the XRD diffraction peaks attributed to Ti_3_C_2_ differs significantly among the samples, with the peak areas of the (104) planes of Ti_3_AlC_2_ for Ti_3_C_2_/TiO_2_ hybrids, Ti_3_C_2_/NH_4_TiOF_3_ (60 °C) and Ti_3_C_2_/NH_4_TiOF_3_ (25 °C) being 252, 270, and 509 arb. Units, respectively. Ti_3_C_2_/TiO_2_ hybrids and Ti_3_C_2_/NH_4_TiOF_3_ (60 °C) have similar amounts of modified MXene; however, the heterojunction of TiO_2_ and Ti_3_C_2_ may have enhanced the photocatalytic activity.

In addition, due to the pseudo-first-order kinetic reaction of MB photodegradation, the apparent rate constants k, calculated based on the experimental data, are approximately 1.2 × 10^−2^, 3.9 × 10^−3^, 2.5 × 10^−3^, and 8.3 × 10^−3^ min^−1^ for Ti_3_C_2_/TiO_2_ hybrids, Ti_3_C_2_/NH_4_TiOF_3_ (60 °C), Ti_3_C_2_/NH_4_TiOF_3_ (25 °C), and anatase TiO_2_ nanoparticles, respectively (Fig. 9(b)). Table 2 compares the apparent rate constants (*k*_app_) for the degradation of organic pollutants using the Ti_3_C_2_/TiO_2_ hybrids investigated in this study with those reported in the literature. The catalytic activity of the Ti_3_C_2_/TiO_2_ hybrids in this study is comparable to or even better than the results obtained in previous studies when MB was considered the target pollutant. Notably, the other Ti_3_C_2_/TiO_2_ MXene-based catalysts showed higher activities against organic compounds other than MB. However, these catalysts require a sintering process at temperatures higher than 120 °C.

**Table 2:** Comparison of apparent rate constants (*k*_app_) for organic pollutants degradation by TiO_2_/Ti_3_C_2_ MXene-based photocatalysts.

Catalyst	*k*_app_ (min^−1^)	Organic pollutants	Synthesis temperature (°C)	Reference
TiO_2_/Ti_3_C_2_ MXene	0.011	Rhodamine B	200 (24 h)	[70]
TiO_2_/Ti_3_C_2_ MXene	0.030	Carbamazepine	160 (12 h)	[71]
TiO_2_/Ti_3_C_2_ MXene	0.019	Phenol	220 (24 h)	[72]
TiO_2_/Ti_3_C_2_ MXene/C_3_N_4_	0.015	Rhodamine B, 4-chlorophenol	120 (12 h)	[73]
NH_4_TiOF_3_	0.0093	MB	250 (2 °C/min)	[74]
Multi-walled carbon nanotubes//TiO_2_	0.014	MB	50 (12 h)	[75]
Ti_3_C_2_/TiO_2_ hybrids	0.012	MB	60 (12 h)	This work

Based on the above photocatalytic results, the synthesized Ti_3_C_2_/TiO_2_ hybrids exhibited at least two properties that contributed to their improved photocatalytic activity. First, the anatase TiO_2_ formed from the NH_4_TiOF_3_ precursor increased the roughness of the TiO_2_ owing to its nanoparticle structure, favoring the adsorption of MB on the surface. In addition, after Ti_3_C_2_ MXene was introduced into the photocatalytic system, the high carrier mobility (2.2 × 10^−5^ cm^2^ V^−1^ s^−1^) of Ti_3_C_2_ MXene enabled a rapid transfer of excited electrons into the Ti_3_C_2_ MXene thin-layer structure, effectively controlling the complex of electron-hole pairs without depositing them on the TiO_2_ surface and generating more active free radicals, which significantly improved the photocatalytic activity of TiO_2_ [76].

**Scheme 2:**
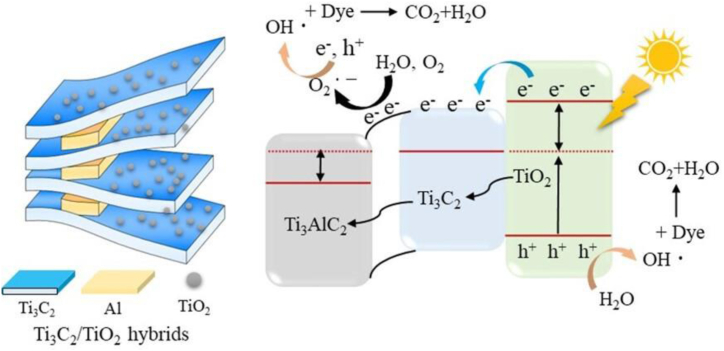
Proposed mechanism of the photocatalysis of methylene blue (MB) over the Ti_3_C_2_/TiO_2_ heterostructure.

The catalytic mechanism of MB decomposition of Ti_3_C_2_/TiO_2_ hybrids is revealed (Scheme 2). The Ti_3_C_2_/TiO_2_ hybrids have Ti_3_C_2_ flakes containing semiconducting TiO_2_ nanocrystals on their surface, leaving an unremoved Ti_3_AlC_2_ MAX phase. A light source loaded with high-energy photons is activated, producing photoinduced electrons (e^–^) in the conduction band of the TiO_2_ component and leaving holes (h^+^) on the valence band. Electrons generated from TiO_2_ can migrate under light irradiation to Ti_3_C_2_ and Ti_3_AlC_2_, which have significantly lower work functions (2.66 eV) than TiO_2_. As a result, Ti_3_C_2_ MXene and Ti_3_AlC_2_ can act as electron reservoirs, suppressing electron-hole pair recombination and prolonging lifetime. After being deposited on Ti_3_C_2_ MXene, the photogenerated electrons move to the surface and react with O_2_ molecules to form superoxide radicals (O_2_^**•**^^–^). The photogenerated holes react with adsorbed hydroxide (OH^−^) molecules to form hydroxyl radicals (•OH). During degradation, the enriched electrons on Ti_3_C_2_ MXene react with H_2_O and O_2_ to form O_2_^**•**^^–^. Finally, radicals with strong oxidizing power (•OH, O_2_^**•**^^–^) break down MB molecules into simple oxidation products.

## 4 Conclusion

In summary, we demonstrated the synthesis of Ti_3_C_2_/NH_4_TiOF_3_ by the exfoliation of Ti_3_AlC_2_ via a two-step process involving (NH_4_)_2_TiF_6_ etching, followed by a hydrolysis reaction with H_3_BO_3_ to produce Ti_3_C_2_/TiO_2_ hybrids. In this study, the exfoliation of Ti_3_C_2_ MXene and simultaneous loading of anatase TiO_2_ nanoparticles onto the surface of layered Ti_3_C_2_ MXene were successfully achieved using the LPD method. The effects of (NH_4_)_2_TiF_6_ concentration, time, and temperature on the etching process were determined. The concentration of the (NH_4_)_2_TiF_6_ etching agent was 1.0 M, the etching time was 24 h, and the temperature was 60 °C. As a preliminary application, the photocatalytic performance of Ti_3_C_2_/TiO_2_ hybrids for the degradation of organic dye molecules was confirmed. The Ti_3_C_2_/TiO_2_ hybrids allowed the high carrier mobility of Ti_3_C_2_ MXene to overcome the problem of the recombination of excited electrons and holes during the TiO_2_ photocatalysis, producing active free radicals and significantly enhancing the photocatalytic activity of TiO_2_. This study reveals a novel and safe approach for the synthesis of Ti_3_C_2_ MXene and homogeneous hybrids of Ti_3_C_2_ MXene and TiO_2_, thereby providing important insights into future applications.

## Author contribution statement

Dr. Tao Wang: Conceived and designed the experiments, performed the experiments, analyzed and interpreted the data, contributed reagents, materials, analysis tools or data; and wrote the paper.

Dr. Li Zhu; Dr. Wanying Zhu: Performed the experiments.

Dr. Hideki Kanda: Conceived and designed the experiments; contributed reagents, materials, analysis tools or data; and wrote the paper.

## Data availability statement

Data will be made available on request.

## Declaration of competing interest

The authors declare that they have no known competing financial interests or personal relationships that could have appeared to influence the work reported in this paper.
